# Formulating Electrolytes for 4.6 V Anode-Free Lithium Metal Batteries

**DOI:** 10.3390/molecules29204831

**Published:** 2024-10-12

**Authors:** Jiaojiao Deng, Hai Lin, Liang Hu, Changzhen Zhan, Qingsong Weng, Xiaoliang Yu, Xiaoqi Sun, Qianlin Zhang, Jinhan Mo, Baohua Li

**Affiliations:** 1 Graphene Composite Research Center, College of Chemistry and Environmental Engineering, Shenzhen University, Shenzhen 518060, China; deng.jiaojiao@szu.edu.cn; 2 Shenzhen Key Laboratory on Power Battery Safety Research and Shenzhen Geim Graphene Center, Tsinghua Shenzhen International Graduate School, Shenzhen 518055, China; woaee2023@gmail.com; 3 Department of Mechanical Engineering and Research Institute for Smart Energy, The Hong Kong Polytechnic University, Hong Kong, China; yu-fm.bai@polyu.edu.hk (C.Z.); qingsong.weng@connect.polyu.hk (Q.W.); xiaoliang.yu@polyu.edu.hk (X.Y.); 4 Department of Chemistry, Northeastern University, Shenyang 110819, China; sunxiaoqi@mail.neu.edu.cn; 5 College of Civil and Transportation Engineering, Shenzhen University, Shenzhen 518060, China; mojinhan@szu.edu.cn

**Keywords:** lithium metal battery, anode free, high voltage, electrolyte, interphase

## Abstract

High-voltage initial anode-free lithium metal batteries (AFLMBs) promise the maximized energy densities of rechargeable lithium batteries. However, the reversibility of the high-voltage cathode and lithium metal anode is unsatisfactory in sustaining their long lifespan. In this research, a concentrated electrolyte comprising dual salts of LiTFSI and LiDFOB dissolved in mixing solvents of dimethyl carbonate (DMC) and fluoroethylene carbonate (FEC) with a LiNO_3_ additive was formulated to address this challenge. FEC and LiNO_3_ regulate the anion-rich solvation structure and help form a LiF, Li_3_N-rich solid electrolyte interphase (SEI) with a high lithium plating/stripping Coulombic efficiency of 98.3%. LiDFOB preferentially decomposes to effectively suppress the side reaction at the high-voltage operation of the Li-rich Li_1.2_Mn_0.54_Ni_0.13_Co_0.13_O_2_ cathode. Moreover, the large irreversible capacity during the initial charge/discharge cycle of the cathode provides supplementary lithium sources for cycle life extension. Owing to these merits, the as-fabricated AFLMBs can operate stably for 80 cycles even at an ultrahigh voltage of 4.6 V. This study sheds new insights on the formulation of advanced electrolytes for highly reversible high-voltage cathodes and lithium metal anodes and could facilitate the practical application of AFLMBs.

## 1. Introduction

Li-ion batteries (LIBs) have dominated the global market for electric vehicles, portable electronics, and grid-scale energy storage because of their high-power output capability, long cycle life, and environmental benignity [1,2,3]. However, the lithium intercalation/de-intercalation electrochemistry of commercially available LIBs results in limited energy densities of less than 300 Wh kg^−1^, which cannot satisfy the growing demand for high-density energy storage systems [4,5,6]. Owing to the ultrahigh theoretical capacity of 3860 mAh g^−1^ and the lowest redox potential of −3.04 V vs. standard hydrogen electrode, metallic lithium anodes have sparked a renewed interest in research towards energy-dense batteries in recent years [7,8]. For instance, lithium metal batteries comprising a lithium metal anode and NCM811 cathode can deliver a high energy density of 300 Wh kg^−1^ [9]. Initial anode-free lithium metal batteries (AFLMB) have been proposed in recent years and comprise a lithium-containing cathode and a bare anode current collector [10,11]. During the first charge process, lithium is plated from the cathode to the anode current collector and then serves as the lithium metal source for subsequent lithium plating/stripping cycles. The electrode weight is minimized, resulting in optimal energy density for the battery. Moreover, the removal of the highly active lithium metal upon cell fabrication makes it much safer and compatible with the LIB production procedure [12].

However, the formation of unstable solid electrolyte interphase (SEI) leads to inevitable lithium loss [13]. It also causes uneven lithium deposition and the generation of “dead” lithium [14]. Both factors result in significant lithium plating/stripping irreversibility. Additionally, lithium dendrites grow during cycling, which could allow them to penetrate the separator and cause short circuits and severe safety concerns [15]. In AFLMBs, the limited lithium source makes their cycle life highly dependent on the lithium plating/stripping reversibility [16]. For instance, a high lithium plating/stripping Coulombic efficiency (CE) of 99.9% sustains a cycle life of 223 cycles at 80% capacity retention of the AFLMBs, which abruptly drops to 5 cycles when the CE decreases to 96%.

Very recently, there has been high demand for rechargeable batteries with super-high energy densities. High-voltage AFLMBs promise maximized energy density [17,18]. Among them, lithium-rich cathode-based AFLMBs could be very attractive since they deliver high reversible capacities of over 220 mAh g^−1^ and a high working voltage of over 4.6 V [19]. Nonetheless, it is still quite challenging to formulate proper electrolytes that can sustain such high-voltage operation while maintaining satisfactory compatibility with the lithium metal anode. Conventional ether electrolytes are compatible with Li anodes but are highly susceptible to oxidative decomposition at high voltages [20,21]. Carbonate electrolytes have high oxidative stability, yet the Li plating/stripping reversibility is unsatisfactory [22,23].

**Figure 1:**
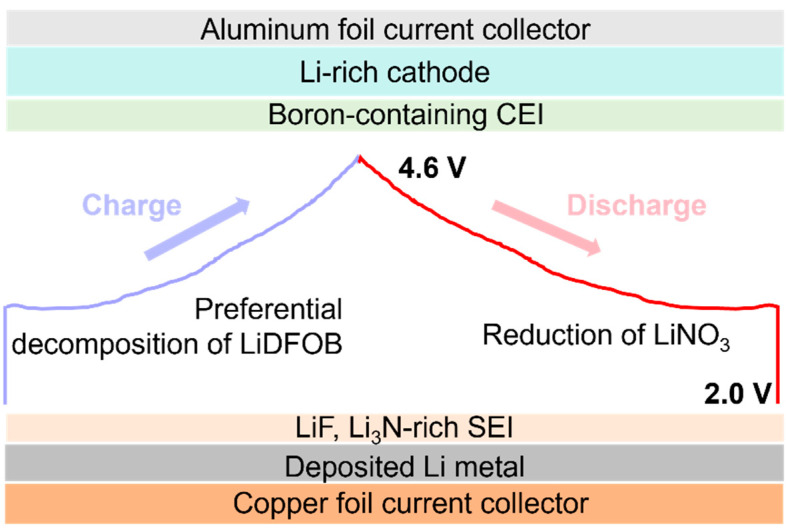
Schematic illustration of electrolyte design for AFLMBs with the Li-rich Li_1.2_Mn_0.54_Ni_0.13_Co_0.13_O_2_ cathode. The blue curve represents the charge profile of AFLMBs with a Li-rich Li_1.2_Mn_0.54_Ni_0.13_Co_0.13_O_2_ cathode, where the LiDFOB additive undergoes preferential decomposition during the charging process. The red curve illustrates the discharge profile, during which the LiNO_3_ additive preferentially decomposes.

In this study, a Li-rich Li_1.2_Mn_0.54_Ni_0.13_Co_0.13_O_2_ cathode was selected to construct a 4.6 V AFLMB with proper electrolyte formulation. Li_1.2_Mn_0.54_Ni_0.13_Co_0.13_O_2_ typically shows an irreversible capacity of over 60 mAh g^−1^ [24], which is disadvantageous in conventional rechargeable batteries but could serve as a supplementary lithium source in AFLMBs for extending the cycle life. A concentrated electrolyte comprising dual salts of lithium bis(trifluoromethanesulfonyl)imide (LiTFSI) and lithium difluoro(oxalato)borate (LiDFOB) dissolved in mixing solvents of dimethyl carbonate (DMC) and fluoroethylene carbonate (FEC) has been formulated. Lithium nitrate (LiNO_3_) dissolved in sulfolane was added to the as-formulated electrolyte. FEC and LiNO_3_ help form a LiF, Li_3_N-rich solid electrolyte interphase (SEI), aiming for a high lithium plating/stripping CE of 98.3%. LiDFOB preferentially decomposes to effectively suppress the side reaction at high-voltage operation of the Li-rich Li_1.2_Mn_0.54_Ni_0.13_Co_0.13_O_2_ cathode (Figure 1). As a result, the as-fabricated AFLMBs can operate stably for 80 cycles even at an ultrahigh voltage of 4.6 V.

## 2. Results and Discussion

**Figure 2:**
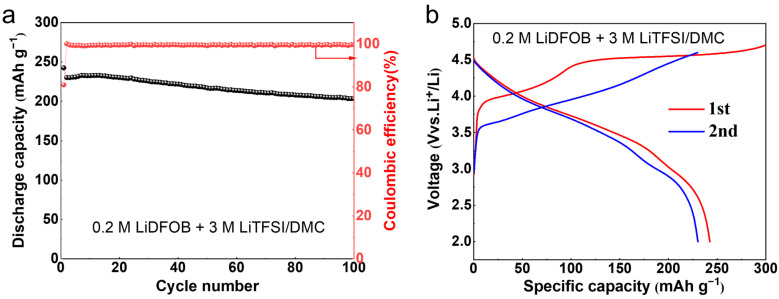
Galvanostatic cyclability (**a**) and charge/discharge curves (**b**) of lithium metal half cells with Li_1.2_Mn_0.54_Ni_0.13_Co_0.13_O_2_ cathode.

The long-term galvanostatic cyclability of the Li_1.2_Mn_0.54_Ni_0.13_Co_0.13_O_2_ cathode was tested in the carbonated-based electrolyte of 3 M LiTFSI 0.2 M LiDFOB/DMC. The initial charge/discharge cycle was performed in the voltage range of 2.0−4.7 V, and the subsequent cycles were conducted in the voltage range of 2.0−4.6 V. The upper voltage limit for the first charge cycle was set to be 4.7 V. The Li_2_O phase from the Li_2_MnO_3_ component of the Li_1.2_Mn_0.54_Ni_0.13_Co_0.13_O_2_ cathode was removed along with the release of O_2_ in the initial cycle [25]. As Figure 2a shows, the cell maintains a discharge-specific capacity of over 200 mAh g^−1^ after 100 cycles at a current rate of 0.5 C. Figure 2b shows the charge/discharge curves in the initial two cycles. It can be observed that the first cycle exhibits a long charging plateau terminating at ~4.6 V, which disappears in the subsequent cycles. This results in a higher specific charge capacity and lower CE in the first cycle. Consequently, the irreversible capacity corresponds to the plated lithium on the anode current collector, which can serve as a supplementary lithium source for subsequent charge/discharge cycles. It can effectively compensate for the active lithium loss at the anode side, thereby extending the cycle life of AFLMBs.

To ensure the long-term cycle life of high-voltage AFLMBs, the irreversible Li loss in each cycle needs to be minimized, and the formation of a cathode electrolyte interphase (CEI) and SEI with minimal electrochemical/mechanical properties is required. It has been reported that Lithium difluoro(oxalate)borate (LiDFOB), as the hybridized form of lithium bis(oxalato)borate (LiBOB) and lithium tetrafluoroborate (LiBF_4_), can be preferentially oxidized at a low potential on the surface of high-voltage cathode materials to generate a dense CEI [20,25,26]. Lithium nitrate (LiNO_3_), a functional additive widely used in ether-based electrolytes for lithium–sulfur batteries, was previously reported to effectively modulate the Li^+^ deposition behavior and form a nitrogen-containing (e.g., Li_3_N and LiN_x_O_y_) SEI layer to stabilize the Li anode [27]. Therefore, LiNO_3_ and LiDFOB salts were introduced into the carbonated-based electrolyte (0.2 M LiDFOB 3 M LiTFSI in FEC/DMC (*v*/*v* = 3:7) with 2.5 wt% 2 M LiNO_3_ in sulfolane, designated as E-LiNO_3_-LiDFOB). Two reference electrolytes of 0.2 M LiDFOB 3 M LiTFSI in FEC/DMC (*v*/*v* = 3:7) (defined as E-LiDFOB) and 3 M LiTFSI in FEC/DMC (*v*/*v* = 3:7) with 2.5 wt% 2 M LiNO_3_ in sulfolane (designated as E-LiNO_3_) were formulated to study the critical role of each component.

**Figure 3:**
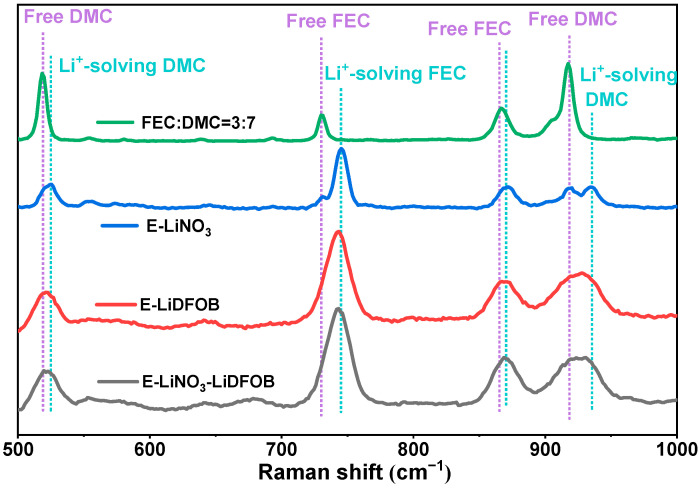
Raman spectra of E-LiNO_3_-LiDFOB, E-LiDFOB, E-LiNO_3_ electrolytes, and FEC/DMC solvents.

To study the solvation structures of three electrolytes, Raman spectra and nuclear magnetic resonance (NMR) measurements were conducted. Figure 3 shows the Raman spectra of E-LiNO_3_-LiDFOB, E-LiDFOB, and E-LiNO_3_ electrolytes and the hybrid FEC/DMC solvent. The peaks at 518 and 917 cm^−1^ are the characteristic peaks of free DMC solvent, and those at 731 and 866.6 cm^−1^ are the characteristic peaks of free FEC solvent. In the E-LiNO_3_ electrolyte, the peak at 731 cm^−1^ shows a blue shift by 14 cm^−1^, and that at 917 cm^−1^ shows a blue shift by 18 cm^−1^ because of the coordination of the FEC/DMC solvents with Li^+^. In the E-LiNO_3_-LiDFOB electrolyte, the blue shift of these peaks is weakened, suggesting that introducing LiDFOB reduces the coordination between FEC/DMC solvents and Li^+^ ions [28].

**Figure 4:**
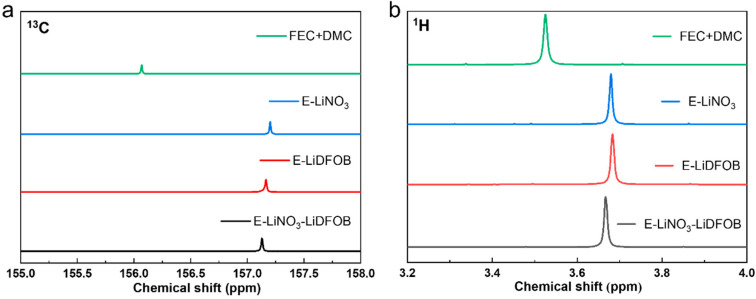
^13^C (**a**) and ^1^H (**b**) NMR spectra of E-LiNO_3_-LiDFOB, E-LiDFOB, and E-LiNO_3_ electrolytes, along with FEC/DMC solvent.

Figure 4 illustrates the ^13^C and ^1^H NMR spectra of E-LiNO_3_-LiDFOB, E-LiDFOB, and E-LiNO_3_ electrolytes and FEC/DMC solvents. When the lithium salts were added to the solvent, the ^13^C and ^1^H chemical shifts of the FEC/DMC solvent changed obviously. In both the ^13^C and ^1^H NMR spectra, larger chemical shifts in the electrolyte are indicative of stronger coordination between Li^+^ ions and the solvent. In comparison to the E-LiNO_3_ electrolyte, the E-LiNO_3_-LiDFOB electrolyte exhibits smaller chemical shifts in both ^13^C and ^1^H NMR spectra. This demonstrates that the addition of LiDFOB additive into the E-LiNO_3_ electrolyte weakens the coordination between the solvent and the Li^+^ ions, thereby altering the electrolyte’s coordination environment and potentially influencing its electrochemical behavior.

**Figure 5:**
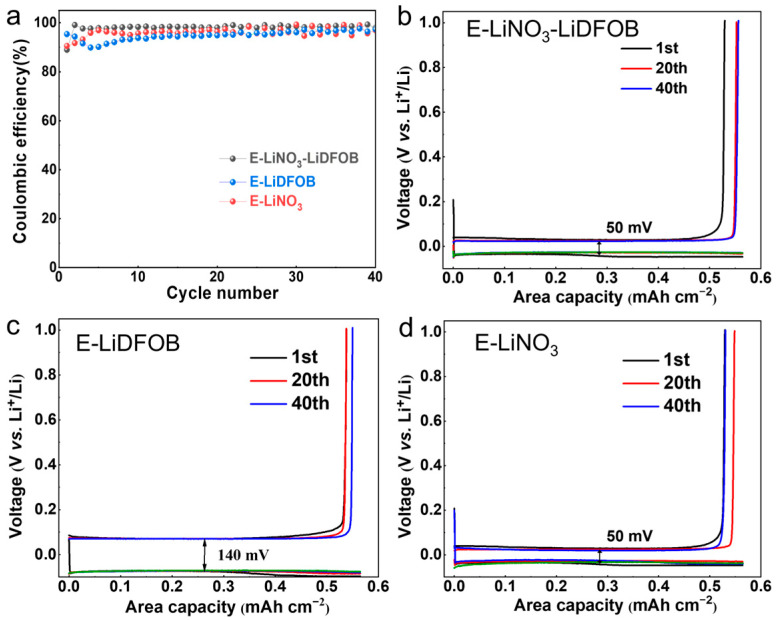
Cycling stability (**a**) of Li plating/stripping cycles and the corresponding charge/discharge profiles with E-LiNO_3_-LiDFOB (**b**), E-LiDFOB (**c**), and E-LiNO_3_ (**d**) electrolytes.

Highly reversible Li plating/stripping is the prerequisite to realizing high-voltage AFLMBs [10,29]. Therefore, the CEs of three electrolytes were evaluated using Li||Cu half-cells. The wettability of electrolytes was examined first. Figure S1 demonstrates that the as-formulated E-LiNO_3_-LiDFOB electrolyte is capable of achieving rapid wetting on aluminum foil and copper foil. As shown in Figure 5a, the Li||Cu half-cell with the E-LiNO_3_-LiDFOB electrolyte shows an average CE of 98.1% over 40 cycles, while that with E-LiDFOB and E-LiNO_3_ electrolytes show an average CE of 94.9% and 96.2%, respectively. The charge/discharge curves of Li plating/stripping cycles in Li||Cu half-cells with three electrolytes were further compared. The Li||Cu half-cells using LiNO_3_-containing electrolytes show a plating/stripping overpotential of 25 mV, which was much lower than 70 mV for those without LiNO_3_ (Figure 5b–d). This indicates that introducing LiNO_3_ to the electrolyte facilitates the kinetics and reversibility of Li plating/stripping. Furthermore, introducing LiDFOB weakens the coordination between FEC/DMC solvents and Li^+^ ions in the E-LiNO_3_-LiDFOB electrolyte, as discussed previously. This weaker coordination facilitates the preferential decomposition of anions and promotes the formation of inorganic-rich SEI, which facilitates higher Li plating/stripping reversibility.

**Figure 6:**
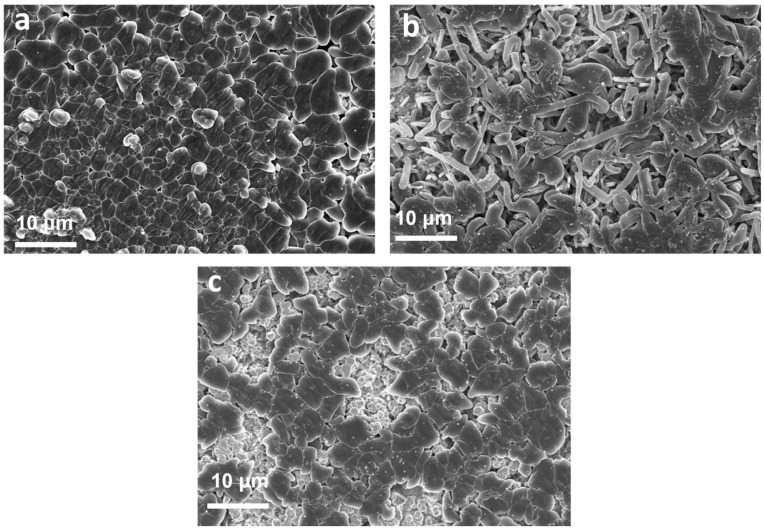
SEM images of the deposited Li metal on Cu foil in Li||Cu half-cells with E-LiNO_3_-LiDFOB (**a**), E-LiDFOB (**b**), and E-LiNO_3_ (**c**) electrolytes after Li plating/stripping cycles at a current density of 0.5 mA cm^−2^ and a capacity of 1 mAh cm^−2^.

The morphology of Li metal deposited on Cu foil after Li plating/stripping cycles in Li||Cu half-cells was studied using scanning electron microscopy (SEM). As shown in Figure 6, the deposited Li metal in cells with E-LiNO_3_-LiDFOB electrolytes exhibited a smooth and uniform morphology, showing large, closely packed grains with minimal voids. In contrast, the deposited Li metal in cells with E-LiDFOB electrolytes revealed a pronounced whisker morphology with many voids. For the E-LiNO_3_ system, no obvious lithium dendrites can be observed. According to the above results, the LiNO_3_ component effectively improves the compatibility between the carbonate-based electrolyte and the Li metal anode, facilitating smooth Li metal deposition, suppressing the formation of Li dendrites, and ensuring high Li plating/stripping reversibility [30,31,32].

**Figure 7:**
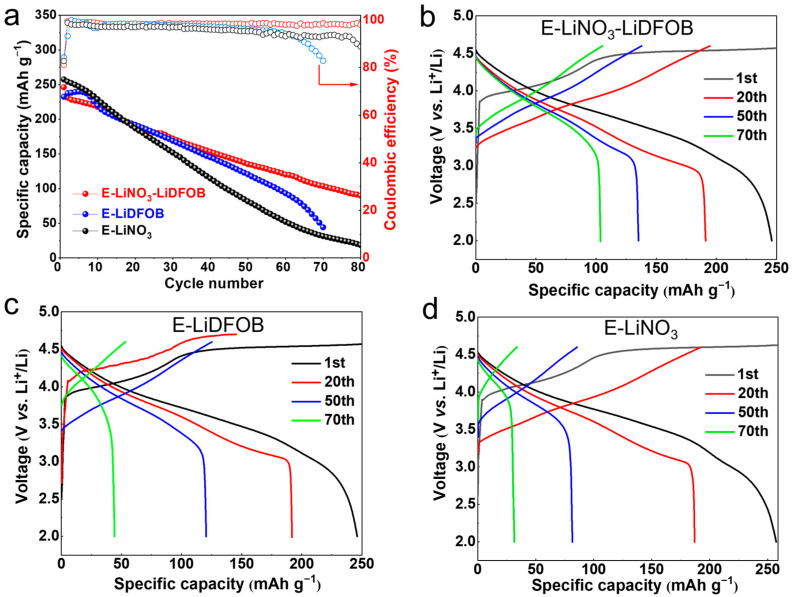
Galvanostatic cyclability (**a**) of high-voltage AFLMB at 0.5 C, and the corresponding charge/discharge profiles with E-LiNO_3_-LiDFOB (**b**), E-LiDFOB (**c**), and E-LiNO_3_ (**d**) electrolytes.

Anode-free full cells with E-LiNO_3_-LiDFOB, E-LiDFOB, and E-LiNO_3_ electrolytes were assembled to study the effects of LiNO_3_ and LiDFOB salts on the high-voltage Li-rich Li_1.2_Mn_0.54_Ni_0.13_Co_0.13_O_2_ cathode. The long-term galvanostatic cyclability and corresponding charge/discharge profiles of AFLMBs with three electrolytes at a current density of 0.5 C are shown in Figure 7. The cells with the E-LiNO_3_-LiDFOB electrolyte and E-LiDFOB electrolyte exhibited a similar capacity decay trend during the first 40 cycles; however, the cells with the E-LiDFOB electrolyte experienced a more rapid capacity decline compared to those with E-LiNO_3_-LiDFOB. The irreversible capacity allows residual lithium metal on the anode to act as a supplementary lithium source for subsequent lithium plating/stripping cycles, decelerating the capacity decay in AFLMBs. The LiNO_3_ component improves the Li plating/stripping reversibility, allowing the limited lithium stored during the first cycle to last over a longer cycling period. Consequently, the cells with the E-LiNO_3_-LiDFOB electrolyte maintain a slower capacity decay over extended cycles than those with the E-LiDFOB electrolyte. Ultimately, the cells with E-LiNO_3_-LiDFOB electrolyte achieve a reversible specific capacity of 90 mAh g^−1^ after 80 cycles, while cells with the E-LiDFOB electrolyte retain less than 50 mAh g^−1^ after 70 cycles. The E-LiNO_3_ system shows a faster capacity decay throughout the charge/discharge cycles compared to those containing the LiDFOB salt. This can be ascribed to LiDFOB decomposition-derived CEI with rich F, B-containing components effectively suppressing the side reaction at the cathode side and facilitating highly reversible lithium transfer between the cathode and anode.

**Figure 8:**
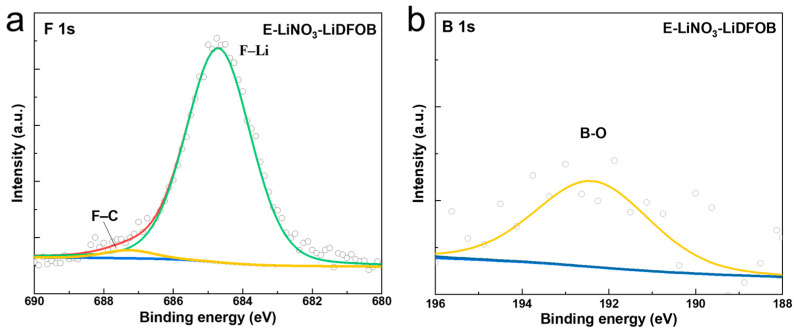
F1s (**a**) and B1s (**b**) spectra of CEI formed on Li-rich Li_1.2_Mn_0.54_Ni_0.13_Co_0.13_O_2_ cathode with E-LiNO_3_-LiDFOB electrolyte.

To further investigate the role of the LiDFOB salt, the compositions of CEI formed on Li-rich Li_1.2_Mn_0.54_Ni_0.13_Co_0.13_O_2_ cathodes with E-LiNO_3_-LiDFOB and E-LiNO_3_ electrolytes after cycling were analyzed using X-ray photoelectron spectroscopy (XPS). Figure 8a and Figure S4 show the F1s spectra of CEI formed in two electrolytes. The CEI formed in the E-LiNO_3_-LiDFOB electrolyte exhibits a dominant F–Li peak at 684.5 eV and a minor F–C peak at 687.5 eV, whereas the F–C peak is much more intense in the E-LiNO_3_ system. This indicates that the LiF component, derived from salt decomposition, is the main constituent of the CEI formed in the E-LiNO_3_-LiDFOB electrolyte. In contrast, the CEI formed in the E-LiNO_3_ electrolyte is primarily derived from the solvent decomposition. Figure 8b shows the B1s spectra of CEI formed in the E-LiNO_3_-LiDFOB electrolyte, with the B–O peak at 192 eV attributed to Li_x_BO_y_F_z_, further confirming that the decomposition of LiDFOB contributes to the formation of an inorganic-rich CEI in the E-LiNO_3_-LiDFOB system.

## 3. Method

### 3.1. Materials

LiDFOB, LiNO_3_, FEC, DMC, and sulfolane were purchased from Sigma-Aldrich, and LiTFSI was ordered from DodoChem. The lithium salts were dried overnight in an argon-filled glovebox (MBRAUN, oxygen < 0.1 ppm, water < 0.1 ppm) before use, and the solvents were treated with 4 Å molecular sieves. Then, 3M LiTFSI and 0.2 M LiDFOB were dissolved in mixed solvents of DMC and FEC (7:3 *v*/*v*). Lithium nitrate LiNO_3_ was dissolved in sulfolane and introduced into the above-prepared electrolyte at 2.5 wt%.

The Li_1.2_Mn_0.54_Ni_0.13_Co_0.13_O_2_ cathode material was purchased from Shenzhen Kejing Co., Ltd. (Shenzhen, China), and used as received. To prepare the cathode film, Li_1.2_Mn_0.54_Ni_0.13_Co_0.13_O_2_ powder, acetylene black (Alfa Aesar Co., Ltd., Shenzhen, China), and polyvinylidene fluoride (PVDF, MTI Co., Ltd., Shenzhen, China) were mixed in the N-methyl pyrrolidinone (NMP, Sigma Aldrich, Shenzhen, China) solvent at a mass ratio of 80:10:10 using a weighing bottle and homogenized by overnight magnetic stirring. The resulting slurry was then spread onto carbon-coated aluminum foil (Al/C, MTI Co., Ltd., Shenzhen, China) using a doctor blade. The obtained cathode film was dried at 120 °C for 6 h in a blast oven and subsequently for 12 h at 120 °C in a vacuum oven. For coin cell assembly, the cathode was prepared by punching discs (12 mm in diameter) and the typical mass loading of the Li_1.2_Mn_0.54_Ni_0.13_Co_0.13_O_2_ active material was ~1.5–2 mg cm^−2^.

### 3.2. Material Characterization

A scanning electron microscope (SEM, HITACHI-SU8220) was used to observe the microstructure and element distribution. Elemental analysis was performed on an energy dispersive X-ray spectroscopy (EDX) spectrometer connected to a HITACHI-SU8220. The electrodes were washed with 1,2-dimethoxyethane and transferred to the SEM chamber using an Ar-filled container before observation. The phase composition was further determined by X-ray diffraction (XRD, SmartLab 9 kW). The X-ray photoelectron spectroscopy (XPS) spectra were measured on a Thermo Scientific spectrometer with an Al-Kα X-ray source. Raman spectra were collected on a Raman spectrum analyzer using a 532 nm laser. ^23^Na-nuclear magnetic resonance (NMR) analysis of the electrolytes was performed using a Jeol ECZ500R 500 MHz Solid-State NMR spectrometer. Prior to the test, dimethyl sulfoxide (DMSO-d6), as a deuterium reagent, was thoroughly mixed with the electrolyte.

### 3.3. Electrochemical Measurements

All electrochemical properties were measured using CR2032 coin cells, which were assembled in an argon-filled glove box with both O_2_ and H_2_O below 0.1 ppm. To evaluate Li plating/stripping efficiency, Li/Cu half cells were assembled using Cu foil as the working electrode (φ16 mm) and Li foil (φ15.5 mm) as the counter/reference electrode. The cells were first cycled five times at 50 μA in the voltage range of 0–1 V (vs. Li^+^/Li), followed by a long-term cycling test at a current density of 0.5 mA cm^−2^ and a lithium deposition capacity of 1 mAh cm^−2^. For the full cell test, anode-free cells comprising the Li_1.2_Mn_0.54_Ni_0.13_Co_0.13_O_2_ cathode and copper current collector as the anode were assembled. The working potential windows in the first charge/discharge cycle and subsequent cycles were 2–4.7 V and 2–4.6 V, respectively.

## 4. Conclusions

In this research, a Li-rich Li_1.2_Mn_0.54_Ni_0.13_Co_0.13_O_2_ cathode was selected to fabricate a 4.6 V AFLMB with proper electrolyte formulation. A concentrated electrolyte comprising dual salts of LiTFSI/LiDFOB dissolved in mixing solvents of DMC/FEC (7:3, *v*/*v*) was formulated. Lithium nitrate (LiNO_3_) dissolved in sulfolane was introduced as a film-forming agent. FEC and LiNO_3_ regulate the anion-rich solvation structure and facilitate the formation of a LiF, Li_3_N-rich solid electrolyte interphase (SEI), aiming for high lithium plating/stripping CE of 98.3%. LiDFOB preferentially decomposes to effectively suppress the side reaction at the high-voltage operation of the Li-rich cathode. Furthermore, the considerable irreversible capacity in the first charge/discharge cycle of the Li_1.2_Mn_0.54_Ni_0.13_Co_0.13_O_2_ cathode offers abundant supplementary lithium sources for cycle life extension. As a result, the as-fabricated 4.6 V AFLMBs can operate stably for 80 cycles with a high specific capacity of 90 mAh g^−1^ maintained.

## Data Availability

The data supporting this study’s findings are available from the corresponding author upon reasonable request.

## Supplementary Materials

The following supporting information can be downloaded at: https://www.mdpi.com/article/10.3390/molecules29204831/s1. Figure S1: Demonstration of the as-formulated E-LiNO_3_-LiDFOB electrolyte wetting the cathode aluminum (a) and anode copper (b) current collectors; Figure S2: Energy dispersive spectroscopy (EDS) mapping of the Li_1.2_Mn_0.54_Ni_0.13_Co_0.13_O_2_ cathode particle; Figure S3: X-ray diffraction (XRD) pattern of the Li_1.2_Mn_0.54_Ni_0.13_Co_0.13_O_2_ cathode and α-NaFeO_2_ structure; Figure S4. F1s of CEI formed on Li-rich Li_1.2_Mn_0.54_Ni_0.13_Co_0.13_O_2_ cathode with E-LiNO_3_ electrolyte; Figure S5. XPS survey scan of CEI formed on Li-rich Li_1.2_Mn_0.54_Ni_0.13_Co_0.13_O_2_ cathode with E-LiNO_3_-LiDFOB electrolyte.
