# Supplementary material for: Formulating Electrolytes for 4.6 V Anode-Free Lithium Metal Batteries
Source: Molecules. 2024 Oct 12;29(20):4831. doi: 10.3390/molecules29204831 (PMC11510016; doi:10.3390/molecules29204831)
Supplement: Supplementary file 1 [file molecules-29-04831-s001.zip › molecules-3234860-supplementary.pdf]

Supporting Information

Formulating Electrolytes for 4.6 V Anode-Free Lithium Metal Batteries

Jiaojiao Deng [1,†], Hai Lin [2,†], Liang Hu [3,*], Changzhen Zhan [3], Qingsong Weng [3], Xiaoliang Yu [3], Xiaoqi Sun [4], Qianlin Zhang [1,*], Jinhan Mo [5] and Baohua Li [2,*]

1 Graphene Composite Research Center, College of Chemistry and Environmental Engineering, Shenzhen Uni-versity, Shenzhen 518060, China; deng.jiaojiao@szu.edu.cn

2 Shenzhen Key Laboratory on Power Battery Safety Research and Shenzhen Geim Graphene Center,
Tsinghua Shenzhen International Graduate School, Shenzhen 518055, China; woaee2023@gmail.com

3 Department of Mechanical Engineering and Research Institute for Smart Energy, The Hong Kong
Polytechnic University, Hong Kong, China; yu-fm.bai@polyu.edu.hk (C.Z.); qingsong.weng@connect.polyu.hk (Q.W.); xiaoliang.yu@polyu.edu.hk (X.Y.)

4 Department of Chemistry, Northeastern University, Shenyang 110819, China; sunxiaoqi@mail.neu.edu.cn

5 College of Civil and Transportation Engineering, Shenzhen University, Shenzhen 518060, China;
mojinhan@szu.edu.cn

* Correspondence: liang3.hu@connect.polyu.hk (L.H.); zhql@szu.edu.cn (Q.Z.); libh@sz.tsinghua.edu.cn (B.L.)

† These authors contributed equally to this work.

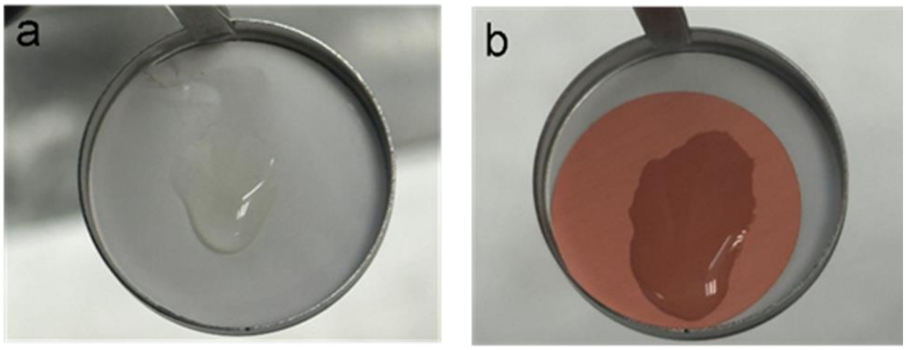

Figure S1. Demonstration of the as-formulated E-$LiNO_3$-LiDFOB electrolyte wetting the cathode aluminum (a) and anode copper (b) current collectors.

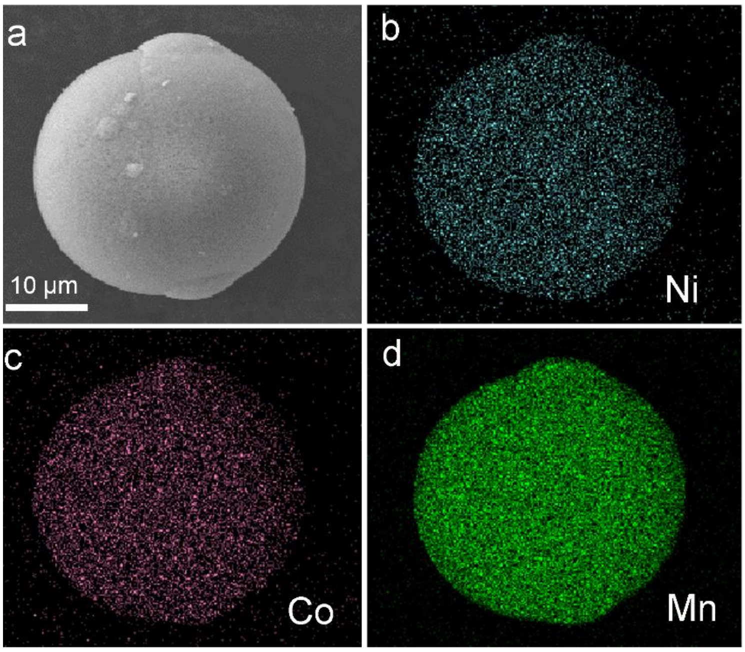

Figure S2. Energy dispersive spectroscopy (EDS) mapping of the $Li_{1.2}Mn_{0.54}Ni_{0.13}Co_{0.13}O_2$ cathode particle.

Energy-dispersive X-ray spectroscopy (EDS) and X-ray diffraction (XRD) analysis were conducted to characterize the Li-rich cathode material. Figure S2a shows that the morphology of Li-rich $Li_{1.2}Mn_{0.54}Ni_{0.13}Co_{0.13}O_2$ cathode material consists of uniform spherical particles with an average diameter of approximately 40 μm. EDS mapping in Figures S2b–d reveals a homogeneous distribution of Ni, Co, and Mn across the spherical particles, confirming the compositional uniformity of the Li-rich cathode.

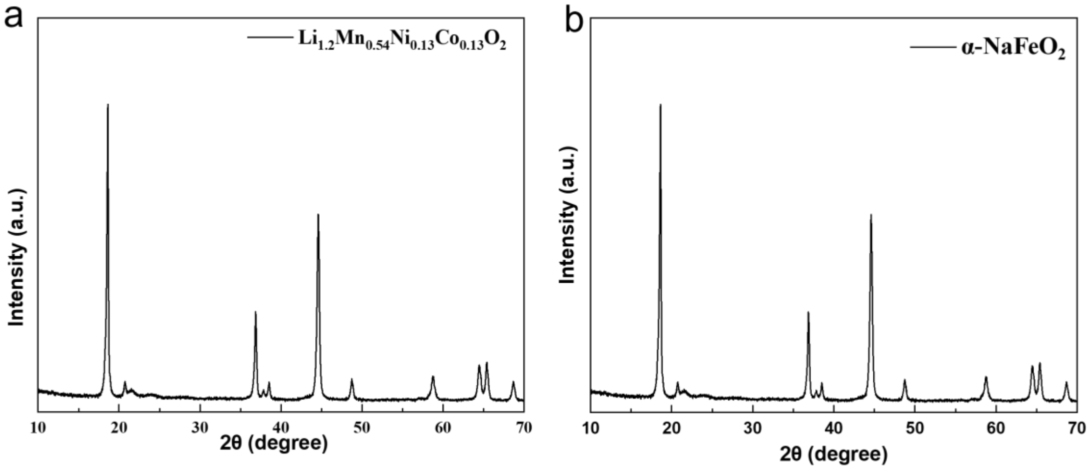

Figure S3. X-ray diffraction (XRD) pattern of the $Li_{1.2}Mn_{0.54}Ni_{0.13}Co_{0.13}O_2$ cathode and α-$NaFeO_2$ structure.

The XRD pattern of the $Li_{1.2}Mn_{0.54}Ni_{0.13}Co_{0.13}O_2$ cathode shows prominent diffraction peaks corresponding to the typical hexagonal α-$NaFeO_2$ structure (PDF-#20-1115) with R-3m space group, which are attributed to the layered $LiMO_2$ component.

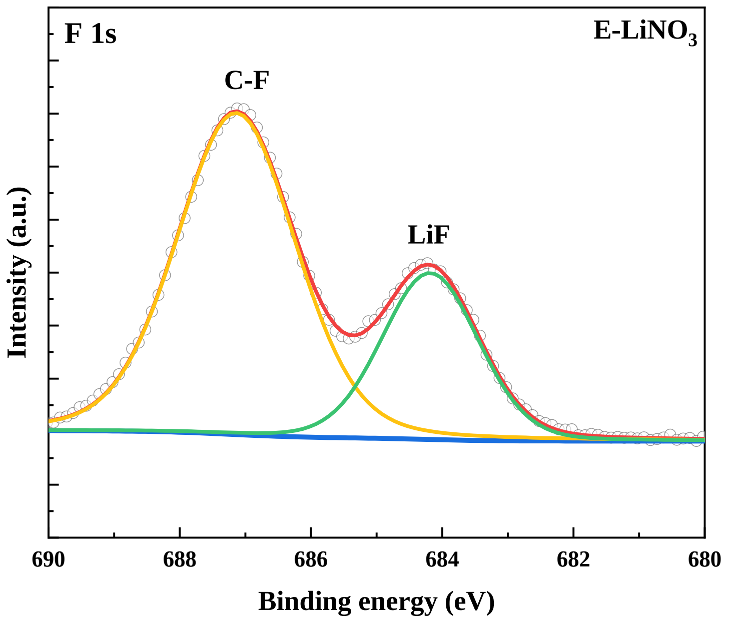

Figure S4. F1s of CEI formed on Li-rich $Li_{1.2}Mn_{0.54}Ni_{0.13}Co_{0.13}O_2$ cathode with E-$LiNO_3$ electrolyte.

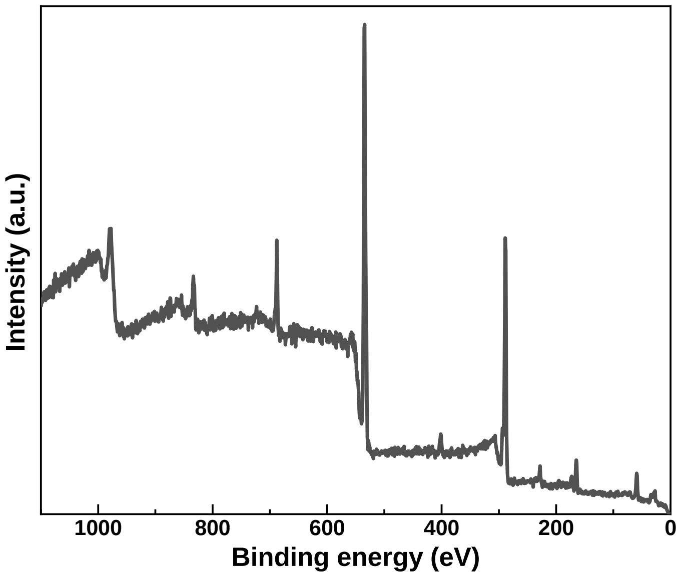

Figure S5. XPS survey scan of CEI formed on Li-rich $Li_{1.2}Mn_{0.54}Ni_{0.13}Co_{0.13}O_2$ cathode with E-$LiNO_3$-LiDFOB electrolyte.
